# Turning premature stop codons into therapeutic opportunities

**DOI:** 10.1016/j.omtn.2025.102825

**Published:** 2026-01-17

**Authors:** James Williamson, Joanna Jacków-Malinowska

**Affiliations:** 1 St John’s Institute of Dermatology & KHP Centre for Translational Medicine, King’s College London, London, UK

## Main text

**Figure 1:**
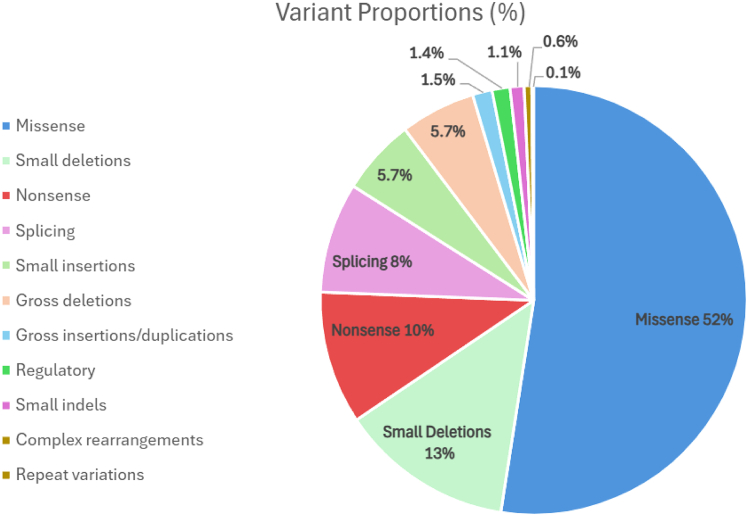
Variant proportions by type Created using data from the Human Gene Mutation Database, a pie chart indicating the proportion of all listed mutations attributed to each type of variant, in descending order of frequency. Nonsense variants, containing a PTC, are indicated to make up 10% of listed, pathogenic variants.

Because genetic diseases arise from many different pathogenic variants, creating a specific genetic therapy for every variant is challenging, leaving most patients with only symptom-focused treatments. However, single-nucleotide, nonsense variants, involving a premature termination codon (PTC), have been identified as 10% of pathogenic variants in genetic disease^1^ (Figure 1) and are found to disrupt tumor suppressor genes in some cancer.^2^ The presence of a PTC leads to the premature termination of translation, causing production of truncated protein, or nonsense-mediated decay of mRNA, preventing expression of functional protein. Drugs that promote translation beyond PTCs, known as translational readthrough-inducing drugs (TRIDs), have the potential to act as variant agnostic therapies, capable of treating patients with diverse PTC variants across multiple diseases. In contrast, gene therapy and gene editing approaches often face skepticism due to their reliance on novel technologies and the permanent nature of the genetic changes they introduce, whereas several TRIDs already approved for clinical use exert only transient, reversible effects.

**Figure 2:**
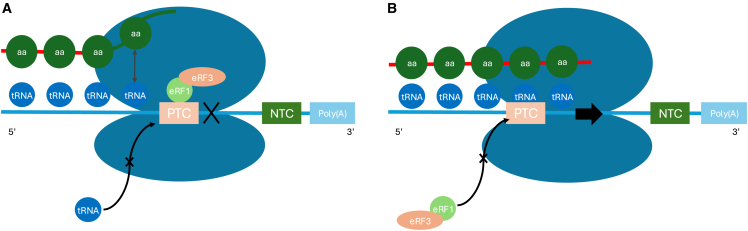
Mechanism of PTC readthrough Translation is shown of an mRNA (light turquoise) containing a premature termination codon (PTC). (A) Translation is terminated through the binding of the PTC by eRF1 and eRF3, interacting with the ribosome (dark turquoise) to prematurely release the polypeptide (dark green) from its tRNA, resulting in a truncated protein. (B) Readthrough occurs by preventing the interaction of eRF1, eRF3, the PTC, and the ribosome. A tRNA instead binds the PTC, allowing continued translation until the next termination codon, the natural termination codon (NTC).

Termination of translation at a termination codon (TC) is a complex process. Unlike amino acid encoding codons, no tRNA perfectly matches TCs; instead, eRF1 recognizes all three TCs and, together with eRF3 and the ribosome, triggers release of the newly synthesized polypeptide (Figure 2).

Occasionally, near-cognate tRNAs (nc-tRNAs) that match two of the three nucleotides can compete with eRF1 at PTCs, allowing insertion of an amino acid and continuation of translation until the next in-frame TC. Readthrough can be promoted by reducing eRF1 or eRF3 activity, disrupting their interaction with the ribosome, or introducing suppressor tRNAs (sup-tRNAs). Because natural termination codons are embedded in sequences that strongly favor termination, PTCs lacking this context are more susceptible to readthrough and therefore more responsive to TRIDs.^3^

TRIDs were first investigated for human disease using the aminoglycoside antibiotic gentamicin to treat cells harboring a cystic fibrosis-causing variant involving a PTC.^4^ It was shown that gentamicin could increase the levels of CFTR, the protein of interest, and that this recovered functionality of the cells. However, gentamicin is limited in its efficacy as higher doses can be toxic, and induce widespread readthrough of natural termination codons (NTCs), leading to a search for safer and more effective drugs.

Miao and colleagues recently investigated the combined use of a novel TRID, CC-90009, with gentamicin as a potential treatment for PTC-related recessive dystrophic epidermolysis bullosa (RDEB) and junctional epidermolysis bullosa (JEB).^5^ In these diseases, PTCs lead to the loss of proteins involved in adherence between the upper and lower layers of the skin, collagen VII (C7) and laminin 332 (Lam332) in RDEB and JEB, respectively. This loss of protein leads to skin fragility, recurrent blistering, and high risk of squamous cell carcinoma. Though each drug separately showed a slight recovery in expression of the relevant protein, when combined, their synergistic action created a greater improvement than simply adding the effects of both drugs together. Further, mimicking a course of treatment by repeatedly dosing cells showed improved protein expression over the time. Staining of the relevant proteins confirmed that they were being correctly localized at the junction between skin layers.

As readthrough brings the concern of producing non-functional protein, assays were employed to investigate the impact of treatment on cellular characteristics. Treated cells showed higher adherence and lower migratory behavior, indicating that the recovered protein was functionally adherent. Additionally, the safety of treatment was considered, with no significant change in cell viability seen after 48 h for treatment. Further, to investigate the effect of therapy on NTC readthrough, the protein Hsp90α was studied, showing no generation of protein above its standard molecular weight, indicating no readthrough of its NTC. Although this is a limited range of safety data, the investigations thus far indicate that combination therapy of CC-90009 and gentamicin could form an effective treatment for both RDEB and JEB, highlighting the value of TRIDs for their capability to treat multiple variants, across diseases.

Further work will be needed to develop this into a clinical therapy, but as with many TRIDs, regulatory barriers may be the greatest challenge. Considering the trajectory of ataluren (Translarna), which held conditional EMA approval for over a decade before its withdrawal after repeated trials that struggled to show significant efficacy, there remains a clear need for continued improvement in TRID efficacy, as well as a re-evaluation of regulation for rare disease therapies.^6^^,^^7^ Encouragingly, recent revisions to the Food and Drug Administration’s (FDA’s) rare-disease framework now allow more flexible evidence requirements and closer collaboration with trial sponsors, creating a clearer path for emerging platforms, including both advanced TRIDs and gene editing therapies.^8^

Indeed, a recent development further strengthens the concept of disease-agnostic approaches by combining gene editing and agnostic PTC targeting. By utilizing prime editors to stably install PTC-specific sup-tRNAs into the genome, the readthrough of PTCs is facilitated in the manner akin to nc-tRNA binding.^9^ Importantly, by endowing cells with the capability to produce their own sup-tRNAs, this technology facilitates the stable, long-lasting capacity to bypass PTCs. This strategy represents a fundamentally different class of variant-agnostic therapy, one that could provide durable correction across many PTC-driven diseases, without the need for repeated re-administration.

Genetically installed readthrough therapies present a route to durable cures, while small molecule TRIDs, as used by Miao et al., may form more accessible therapies, as “off the shelf,” oral delivery, comparable to Translarna. This approach may increase availability of treatment in areas without the expertise and resources needed for cell or gene therapy and could also offer a low treatment burden for patients, important in diseases such as RDEB and JEB where treatment requires a significant commitment. These diverse applications of PTC-targeting treatments allow them to be more than placeholders for gene correction, but viable therapies in their own rights. Realizing this potential of PTC readthrough will require coordinated effort between regulators, researchers, and clinicians, but the shift toward more accommodating regulatory pathways offers a promising opportunity to bring truly transformative treatments to patients who currently have no alternatives.

## Acknowledgments

We are grateful for the support of CureEB, DEBRA UK, EB Research Partnership & EB Medical Research Foundations (“EB Charities”), 10.13039/501100000296British Skin Foundation, Rosetree Foundation, and EI Cure Project to J.J.-M.

## Author contributions

J.W. and J.J.-M. contributed equally to conception of ideas and writing.

## Declaration of interests

The authors declare no conflicts of interest.
